# Multi-omics differentially classify disease state and treatment outcome in pediatric Crohn’s disease

**DOI:** 10.1186/s40168-018-0398-3

**Published:** 2018-01-15

**Authors:** Gavin M. Douglas, Richard Hansen, Casey M. A. Jones, Katherine A. Dunn, André M. Comeau, Joseph P. Bielawski, Rachel Tayler, Emad M. El-Omar, Richard K. Russell, Georgina L. Hold, Morgan G. I. Langille, Johan Van Limbergen

**Affiliations:** 1 Department of Microbiology and Immunology, Dalhousie University, Halifax, NS Canada; 2 Department of Paediatric Gastroenterology, Royal Hospital for Children, Glasgow, UK; 3 Department of Pharmacology, Dalhousie University, Halifax, NS Canada; 4 Department of Biology, Dalhousie University, Halifax, NS Canada; 5 CGEB-Integrated Microbiome Resource (IMR), Dalhousie University, Halifax, NS Canada; 6 Department of Medicine, St George and Sutherland Clinical School, UNSW, Sydney, NSW Australia; 7 Department of Pediatrics, Dalhousie University, Halifax, NS Canada

**Keywords:** Crohn’s disease, Treatment response, Machine learning, Microbiome, Treatment-naïve, Pediatric

## Abstract

**Background:**

Crohn’s disease (CD) has an unclear etiology, but there is growing evidence of a direct link with a dysbiotic microbiome. Many gut microbes have previously been associated with CD, but these have mainly been confounded with patients’ ongoing treatments. Additionally, most analyses of CD patients’ microbiomes have focused on microbes in stool samples, which yield different insights than profiling biopsy samples.

**Results:**

We sequenced the 16S rRNA gene (16S) and carried out shotgun metagenomics (MGS) from the intestinal biopsies of 20 treatment-naïve CD and 20 control pediatric patients. We identified the abundances of microbial taxa and inferred functional categories within each dataset. We also identified known human genetic variants from the MGS data. We then used a machine learning approach to determine the classification accuracy when these datasets, collapsed to different hierarchical groupings, were used independently to classify patients by disease state and by CD patients’ response to treatment. We found that 16S-identified microbes could classify patients with higher accuracy in both cases. Based on follow-ups with these patients, we identified which microbes and functions were best for predicting disease state and response to treatment, including several previously identified markers. By combining the top features from all significant models into a single model, we could compare the relative importance of these predictive features. We found that 16S-identified microbes are the best predictors of CD state whereas MGS-identified markers perform best for classifying treatment response.

**Conclusions:**

We demonstrate for the first time that useful predictors of CD treatment response can be produced from shotgun MGS sequencing of biopsy samples despite the complications related to large proportions of host DNA. The top predictive features that we identified in this study could be useful for building an improved classifier for CD and treatment response based on sufferers’ microbiome in the future.

The BISCUIT project is funded by a Clinical Academic Fellowship from the Chief Scientist Office (Scotland)—CAF/08/01.

**Electronic supplementary material:**

The online version of this article (10.1186/s40168-018-0398-3) contains supplementary material, which is available to authorized users.

## Background

Crohn’s disease (CD) is an inflammatory bowel disease (IBD) classically characterized by abdominal pain, rectal bleeding and weight loss. Recurring flares of IBD cause lifelong, far-reaching consequences for patients that can affect lifestyle and overall health [1, 2]. CD differs from the other form of IBD—ulcerative colitis—in that CD can affect any part of the gastrointestinal tract, can be discontinuous, and can involve granulomatous inflammation [3]. There is a growing need to understand the etiology of CD due to the worldwide increase in annual incidence [4], particularly in children [5].

Although the etiology of CD is unclear [6], there is growing evidence for the dysbiosis hypothesis. This model postulates that a shift in the balance between commensal and pathogenic intestinal microbes interacting with the host’s immune system contributes to CD onset. In support of this model, large-scale differences in bacterial abundances have long been associated with CD [7]. The most reproducible finding has been a decrease in alpha-diversity in CD patients compared to controls [8–10]. Several particular changes in taxonomic abundances have been linked to this dysbiotic state; for instance, Firmicutes tend to be at lower proportion and Gammaproteobacteria at higher proportion in CD patients [11]. Most taxonomic profiles of CD patients have been based on stool samples, which yield drastically different insights into CD pathogenesis when compared with mucosal washing of the mucosal-luminal interface (MLI) and intestinal biopsy samples [8]. Irrespective of body site, it is unclear whether these shifts in microbiota are a cause or a symptom of the disease. However, there is reason to believe that the microbiome contributes to CD etiology due to several observations. Firstly, children that are exposed to antibiotics in the first year of life are more likely to develop IBD [12], which could be related to acquiring a dysbiotic state. Also, many CD risk loci are linked to pattern recognition receptors (PRRs) and cytokines that regulate the host immune system [13].

PRRs generate responses against pathogenic bacteria while identifying commensal bacteria within the human microbiome. The best-known example of a PRR linked to CD is the nucleotide-binding oligomerization domain containing protein 2 (*NOD2*) gene that codes for an intracellular PRR. Loss of function mutations in the gene lead to increased inflammation due to impaired clearance of intestinal bacteria that are harmful to the gut [14]. Despite these reproducible links to CD, risk mutations account for < 14% of disease variance across patients [15]. However, the concordance rate of CD between monozygotic twins ranges from 20 to 50%, which is higher than several other complex diseases [16]. Nonetheless, risk loci alone do not explain CD onset and the relative importance of the microbiome in the onset of this disease is not well understood.

Here, we compare the relative importance of genetic risk loci and microbiota identified from intestinal biopsy samples for classifying treatment-naïve pediatric patients by disease state. We also demonstrate that CD patients’ treatment response status can be classified by microbial features with high accuracy. Taxonomic and functional profiles discussed in this study are based on both 16S sequencing and metagenomics (MGS) sequencing of the same intestinal biopsy samples. To our knowledge, this is the first report of shotgun MGS of CD intestinal biopsy samples.

## Methods

### Sequenced samples

Intestinal biopsies were previously taken from 20 Crohn’s disease (CD) and 20 normal colon control patients as part of the “Bacteria in Inflammatory bowel disease in Scottish Children Undergoing Investigation before Treatment” (BISCUIT) cohort [9, 17]. We did not perform a power test to predict what effect sizes could be detected with this sample size, but instead chose this sample size due to sequencing cost constraints. These patients were all under 17 years old with a mean age of 12.7 years. CD biopsies were obtained at the diagnostic endoscopy prior to commencing any therapy. We based CD diagnosis on the Paris Classification [18]. None of these patients used systemic antibiotics or steroids in the 3 months prior to their colonoscopy or immunosuppression at any point. Treatment response was classified as sustained remission following induction treatment response and was defined by physician global assessment and the requirement for treatment escalation (repeat induction therapy) before 24 weeks.

### Metagenomics sequencing and bioinformatic pipeline

Shotgun MGS preparation and sequencing was conducted by Génome-Québec (McGill University, Montréal, Québec) on an Illumina HiSeq. A mean of 110 million PE 100 base pair (bp) MGS reads were produced with a range of 72.7–135 million reads over all samples. We first concatenated FASTQ files containing forward and reverse reads into a single FASTQ per sample. We then screened out contaminant sequences by mapping all reads against the human (hg19) and PhiX (RTA) genomes using bowtie2 [19] (v2.2.6), which resulted in a mean of 90% of reads being excluded. This high percentage of contaminant reads is mainly due to the high proportion of human cells in biopsy samples, which is less of an issue for microbiome studies that focus on stool samples. After screening out these non-microbial reads, we classified the remaining reads taxonomically using MetaPhlAn2 [20] (v2.2.0) with the “–very-sensitive” global alignment option and into KEGG orthologs (KOs) using HUMAnN2 [21] (v0.11.1; http://huttenhower.sph.harvard.edu/humann2). Importantly, we found that running bowtie2 in local alignment mode with MetaPhlAn2 resulted in many spurious hits, which were mainly represented by viruses. These taxa were not identified when global alignment was performed. We ran MUSiCC [22] (v1.0.2) to normalize the KO abundances within each sample by the median universal single-copy gene abundance, which controls for inter-sample variation in microbial genome sizes. We then ran HUMAnN2 on these normalized values to reconstruct KEGG module and pathway abundances within each sample. No taxa or functions were identified in the MGS of two samples, S34 and S38 (16S sequencing also failed for these samples, see below), which were excluded from downstream microbiome analyses.

### Calling human variants

Due to the large percentage of human DNA in our MGS (see above), we were also able to call human variants from the same dataset. Although we used 133 loci for calculating the genetic risk score (see below), we called genome-wide variants to improve imputation accuracy in cases where samples were missing data at these sites. We began by mapping all MGS reads to the human genome (hg19) using the Burrows-Wheeler Alignment Tool’s [23] (v0.7.12) mem algorithm, which resulted in a 98% mapping rate. This mapping rate is higher than the rate for the metagenomic microbial pipeline due to the different algorithms used for each workflow. We then followed the Genome Analysis ToolKit’s (GATK) [24] Best Practices workflow [25, 26] for variant calling. Pre-processing steps included marking duplicate reads, recalibrating base quality scores based on a model trained on known variants, and re-aligning reads around known insertions and deletions. We then ran the GATK (v3.5) program HaplotypeCaller to call variants using default parameters and variant quality score recalibration per the Best Practices workflow. These steps resulted in 16,333,869 raw variants based on a genome-wide mean coverage of 7.5 reads across all 40 individuals. Due to the low genome-wide coverage, we also discarded variants based on several hard filters implemented by VCFtools [27] (v0.1.13): any variant not in Hardy-Weinberg equilibrium (cut-off significance of *P* < 1×10^−4^), any variant called by <6 reads, or any variant with >50% missing data. We retained 7,604,626 variants following these hard cut-offs. The 133 known risk loci were not required to pass these hard cut-offs.

### Imputing missing genotypes

After calling variants genome-wide, we next imputed the missing genotypes for the 133 known CD risk loci. Three variants (rs9264942, rs11209026, rs6927022) were missing genotype calls in all samples and were excluded. Haplotype phasing and the first pass of imputation were performed with SHAPEIT [28] (v2.r837). IMPUTE2 [29, 30] (v2.3.2) was then run on SHAPEIT’s phased output to impute the final genotypes. The HapMap phase II b37 genetic map was used for both imputation steps, and the 1000 Genomes Phase 3 [31] phased haplotypes were used as reference haplotypes. Default parameters were used for running both SHAPEIT and IMPUTE2.

### Genetic risk scores

A custom Perl script was used to parse the IMPUTE2 output into a variant call format, and then PLINK [32] (v1.90b3.29) was used to convert this table into PED and MAP files. Per-sample genetic risk scores (GRS) were calculated using the Mangrove R package [33]. To calculate the GRS, we used the genotypes at these imputed risk loci, odds-ratio information for risk alleles, and minor allele frequencies from previously published genome-wide association studies [15, 34]. We assumed a CD prevalence of 1% when calculating GRS (*K* value = 0.01).

### 16S rRNA gene sequencing

The intestinal biopsy samples were prepared for 16S sequencing using our Microbiome Amplicon Sequencing Workflow [35]. Briefly, the pre-extracted DNA [17] was first amplified in duplicate using dual-indexing Illumina primers (forward: ACGCGHNRAACCTTACC; reverse: ACGGGCRGTGWGTRCAA) that targeted the V6-V8 region (438 bp) of the bacterial 16S rRNA gene. The pooled duplicate PCR products were verified using high-throughput E-gels (Invitrogen), then purified and normalized using the SequalPrep 96-well Plate Kit (Invitrogen). Following quantification, the pooled samples were run on an Illumina MiSeq using PE 300 + 300 bp v3 chemistry at the Integrated Microbiome Resource (Dalhousie University, Halifax, Nova Scotia).

### 16S rRNA gene bioinformatic pipeline

We followed the Microbiome Helper standard operating procedure [35] to process the 16S rRNA gene data. Two CD samples (S34 and S38) were excluded from this pipeline due to low DNA quality and repeated sequencing failures, which left a total of 38 samples remaining (20 CN and 18 CD). A mean of 21,793 raw PE read pairs were produced over these remaining samples (min = 9503; max = 40,392). Forward and reverse reads were then stitched together using PEAR [36] (v0.9.6) with an assembly rate > 80% for all samples except for sample S22 (68.7% of reads assembled). We then filtered out stitched reads with a quality score < 30 over 90% of bases using the FASTX toolkit (v0.0.14; http://hannonlab.cshl.edu/fastx_toolkit/). We also filtered out reads < 400 bp or that did not have exact matches to the forward and reverse primers using BBMap (v35.82; https://sourceforge.net/projects/bbmap/). An average of 18.7% of the assembled reads per sample was discarded by these filters. Next, we removed chimeric sequences using UCHIME [37] (v6.1) with the parameters mindiv = 1.5 and minh = 0.2, which resulted in an average of 16.3% of the assembled reads being discarded. Following these filters, a mean of 13,815 reads were remaining per sample (min = 4427; max = 27,472). We ran open-reference 97% OTU picking using QIIME (v1.9.0) wrapper scripts with these filtered reads. Reference OTU picking was run against the Greengenes [38] (v13_8) database using SortMeRNA [39] (v2.0-dev, 29/11/2014) with a minimum query coverage of 80% and de novo OTU picking using SUMACLUST (v1.0.00; https://git.metabarcoding.org/obitools/sumaclust/wikis/home/). We filtered out OTUs that were called by < 0.1% of reads and then rarefied read counts to 4000 reads per sample, which resulted in a final set of 984 OTUs. PICRUSt [40] (v1.0.0) was used to predict KEGG ortholog and pathway abundances based on reference OTU abundances. We compared the rarefied OTU abundances to non-rarified abundances after performing a centered log-ratio transformation [41]. Read counts were imputed with the count zero multiplicative method in the zCompositions R package [42] (v1.1.1) before performing the centered log-ratio transformation. We compared these workflows by evaluating how well models performed using abundance tables produced by each workflow. To evaluate concordance between MGS and 16S-identified genera, we calculated the Spearman’s correlation (*ρ*) of the relative abundances of 16S genera at greater than 10% frequency and identified in both datasets.

### RISK validation cohort

We downloaded single-end sequencing of the V4 region of the 16S gene produced for the “Risk Stratification and Identification of Immunogenetic and Microbial Markers of Rapid Disease Progression in Children with Crohn’s Disease” (RISK) cohort [8] from the National Center for Biotechnology Information under study accession PRJEB13679. We reduced this data to 773 biopsy samples that were either controls or CD patients and ≤ 18 years old. To process this data, we first merged together sequencing replicates for the same samples. We then trimmed all reads to 130 nucleotides using Trimmomatic [43] (v0.36). The remaining steps were the same as the 16S processing pipeline described above. The OTU table was rarefied to 4000 reads (42 samples with depth below this cut-off were discarded), which resulted in 2564 OTUs being called over 731 samples.

### Random forest classification

For each dataset, we ran random forest (RF) models to classify disease state and treatment response separately. Each dataset was pre-processed, so only features with > 10% non-zero values were retained. Each table was then standardized by sample (subtracted the sample’s mean and then divided by the sample’s standard deviation). We ran RF models using the random forest [44] (v4.6.12) R package with default *mtry* values and used 712 as the random seed. All models were run with 10,001 trees except for the KO models which were run with 501 trees to reduce running time. RF model significance was determined by the permutation test implemented in the rfUtilities [45] (v2.0.0) R package. This test involves building a null distribution of out-of-bag (OOB) errors from RF models with randomized classes (e.g., the disease state column of the input table was randomized). Model significance is then determined by calculating whether < 5% of random permutation models have an OOB error less than or equal to the observed OOB error. Significance of RF models as tested by the above permutation procedure was treated as an omnibus test for any association between the signal derived from genetic data and the feature labels of each sample. This allowed us to identify at what level (e.g., family, genus and species) further investigation was warranted and supported our investigation of variable importance in some “datasets” and not others. Note that RF models make no assumptions about how the input features are distributed. Leave-one-out cross-validation was also run on each dataset to output accuracy for each model with the R package caret [46] (v6.0.77).

## Results

### Identifying CD-related SNPs, microbial taxa, and functions from intestinal biopsy samples

**Fig. 1:**
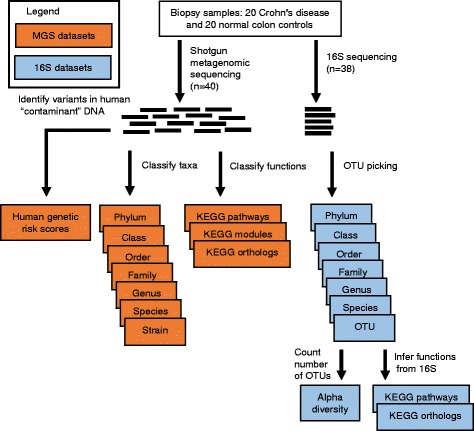
Diagram of the different datasets used for classification in this study. Datasets in orange were derived from the shotgun metagenomic sequencing (MGS) data (*n* = 40) and the datasets in blue were derived from the 16S rRNA gene (16S) sequencing data (*n* = 38*). These datasets were used to classify both disease state and treatment response as input to random forest machine learning models. *Note two Crohn’s disease samples were removed from both the 16S sequencing and MGS datasets due to low sequencing coverage, but their genetic profile was inferred from the MGS

To investigate which microbial and genetic features best classify pediatric CD patients by disease state and treatment response, we sequenced the intestinal microbiomes of 20 CD and 20 normal colon controls prior to any treatments. Both MGS and 16S sequencing were performed on the same biopsy samples. Much of the MGS data was comprised of human DNA (90%), which was separated from the microbial DNA and used to call human genotypes. We combined the human genotypes at 133 known CD risk loci with known odds-ratios and allele frequencies to calculate a genetic risk score [33] (GRS) per sample. We then used the remaining microbial MGS reads, a mean of 10.7 million paired-end (PE) reads per sample, to call 115 independent taxa (summarized at the class level in Additional file 1: Figure S1). All microbial MGS reads were also used to identify the relative abundances of Kyoto Encyclopedia of Genes and Genomes [47] (KEGG) orthologs, pathways, and modules within each sample. Similarly, after filtering the 16S amplicon reads, we retained an average of 13,815 stitched reads per sample. We performed open-reference clustering to call 984 operational taxonomic units (OTUs; summarized at the class level in Additional file 1: Figure S2). Overall, the relative abundances of MGS and 16S-identified genera were similar within the same biopsy samples (mean Spearman’s *ρ* = 0.51, standard deviation = 0.18). Since sequencing read counts are a form of compositional data, we tested whether a centered log-ratio transform of the non-rarefied read counts [41] would result in improved model performance compared to rarefaction of all samples. Although the compositional-based methods performed slightly better for some feature tables, in the majority of cases, this transformation resulted in less accurate classification of patients (see below; Additional file 1: Figure S3), and so, we focused on the rarefied datasets for our analyses. We used these OTUs to infer the relative abundances of KEGG orthologs and pathways within each sample (see Additional file 2 for sample sequencing coverage and metadata). Two of the CD patients’ microbial profiles were discarded due to low 16S and MGS sequencing depth. These different datasets are outlined in Fig. 1 (see Additional file 1: Table S1 for sample details).

We then replicated two well-known predictors of CD: increased GRS [1] and a reduction in microbial alpha-diversity as proof of principle. We chose the simplest measure of alpha-diversity: the observed number of OTUs per sample (# OTUs). Both GRS (one-tailed Mann-Whitney-Wilcoxon (M-W-W) test, *W* = 288, *P* = 0.00837) and # OTUs (one-tailed M-W-W test, *W* = 261.5, *P* = 0.00894) significantly differed between patients based on disease state in the expected directions (Additional file 1: Figure S4). To make these known predictors comparable to classification accuracies using datasets containing multiple features, we used an analogous method to calculate accuracy. Importantly, these metrics produced only marginal accuracies when used to classify patients by disease state (GRS, 62.5%; # OTUs, 71.1%).

### Classifying samples by disease state

**Fig. 2:**
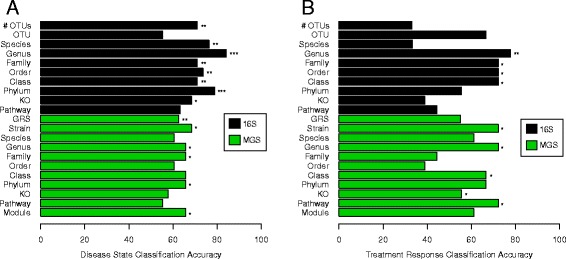
Classification accuracies for all datasets classifying **a** disease state and **b** treatment response. Each bar corresponds to a different model. Accuracies are based on random forest (RF) leave-one-out cross-validation (LOOCV) in all cases, except for number of observed OTUs (# OTUs) and genetic risk scores (GRS) which are based on LOOCV of simple linear cut-off models. The symbols *, **, and *** indicate significance at *P* < 0.05, *P* < 0.01, and *P* < 0.001, respectively. RF model significances were based on a permutation test. *P* values for # OTUs and GRS are based on one-tailed Mann-Whitney-Wilcoxon Tests

We next investigated how well microbial datasets classify CD disease state. MGS and 16S taxonomic datasets included strain and OTU-level-relative abundances respectively and were also collapsed at each level from species to phylum (Fig. 1). Functional datasets included KEGG ortholog and pathway counts for both sequencing technologies, as well as KEGG modules for MGS samples. In total, 19 datasets were entered as classifiers for disease state after standardization (each mean-centered and scaled by the standard deviation for each sample). We ran independent random forest (RF) models to determine each dataset’s classification accuracy (Fig. 2a; see Additional file 3). Each of the 16S taxonomic datasets, except for the OTU level, could classify patients by disease state with high accuracy (maximum accuracy of 84.2% and *P* < 0.001 based on genus level). The MGS strain, genus, family, and phylum taxonomic datasets also classified patients, but with lower accuracy than the 16S datasets (maximum accuracy of 68.4% and *P* = 0.016 based on strain level). The predicted KO abundances based on the 16S data and the MGS-identified KEGG modules both significantly classified patients as well (accuracies of 68.4 and 65.8% respectively).

One advantage of RF models is that they output variable importance metrics for each feature used in a model. We considered each RF model to be an omnibus test for each dataset, which enabled us to look at the ranking of variable importance in significant models to identify important features (see Additional file 4). Based on these metrics, the three most informative 16S genera were *Desulfovibrio*, *Akkermansia*, and *Butyricimonas* (Additional file 1: Figure S5), whereas the top MGS genera were *Alistipes*, *Oscillibacter*, and *Dorea*. These top genera could differ since both top 16S genera were close to the detection limit threshold of the MGS data; they were only identified in a small number of samples (Additional file 1: Figure S6). Nonetheless, *Akkermansia* was ranked fourth in the MGS genus model despite being missed in several samples. The top features in the MGS strain model were strains of *Alistipes putredinis*, *Clostridium symbiosum*, and *Faecalibacterium prausnitzii*. The 16S-inferred KOs, and the MGS modules were the only functional datasets that significantly classified samples by disease state (accuracy = 68.4%, *P* = 0.043 and accuracy = 65.8%, *P* = 0.03, respectively). The three top 16S KOs were (1) K03785, which is involved in amino acid biosynthesis, (2) K09013, an Fe-S cluster assembly ATP-binding protein, and (3) K03809, a tryptophan repressor binding protein. The three top MGS modules were (1) M00144, NADH: quinone oxidoreductase, (2) M00362, nucleotide sugar biosynthesis, and (3) M00239, peptides/nickel transport system. Importantly, the datasets collapsed to different taxonomic and functional levels were not independent from each other, which is reflected by the fact that the top features in each taxonomic dataset tended to be part of the same lineage (e.g., the ranks above *Desulfovibrio* and *Akkermansia* were also top hits).

### Classifying samples by treatment response

Next, we used these same 19 microbial datasets, after excluding normal colon control patients, to classify the CD patients as responders (RS) and non-responders (NR) to induction of remission treatments, started at the time of diagnosis (Fig. 2b; see Additional file 3). Clinical CD phenotypes were heterogeneous, but all included active colonic disease at the sampled location. Treatments were similarly not consistent across all patients, reflecting heterogeneity of phenotype, but instead were different combinations of exclusive enteral nutrition (EEN) therapy and immunosuppressive medications, as such representing ‘real-world’ CD treatment: 11 patients were on EEN, 3 were on prednisolone and EEN therapy, 4 were on mesalazine alone, and 2 were on prednisolone alone, as decided by their gastroenterologist at the time of diagnosis. Sustained response or non-response was defined as need for a second induction within 150 days of diagnosis or not (Additional file 1: Table S2). After classifying CD patients based on their response to induction treatment, 16S genera were again the top dataset (accuracy = 77.8%; *P* = 0.008). However, the MGS strain (*P* = 0.029), genus (*P* = 0.013), and KEGG pathway (*P* = 0.018) datasets could also classify patients with only slightly lower accuracy (accuracy = 72.2% for all three). We also found that alpha-diversity and GRS did not significantly differ between RS and NR patients (Additional file 1: Figure S7).

Using the same omnibus test approach as above, we were again able to identify the most informative features in each significant dataset (see Additional file 5). The top 16S genera were *Dialister*, *Bilophila*, and *Aggregatibacter* in this analysis. The top MGS strains were subtypes of *Parabacteroides merdae*, *Sutterella wadsworthensis*, and an unclassified strain within the Lachnospiraceae family. The top MGS genera included *Parabacteroides*, *Bacteroides*, and an unclassified genus of Lachnospiraceae. The top MGS KEGG pathways included (1) ko00633, nitrotoluene degradation; (2) ko00250, alanine, aspartate, and glutamate metabolism; and (3) ko00230, purine metabolism. The top KOs were (1) K02954, a ribosomal protein, (2) K07259, which is involved in peptidoglycan biosynthesis, and (3) K07793, a putative tricarboxylic transport membrane protein.

### Comparing the relative importance of top features

**Fig. 3:**
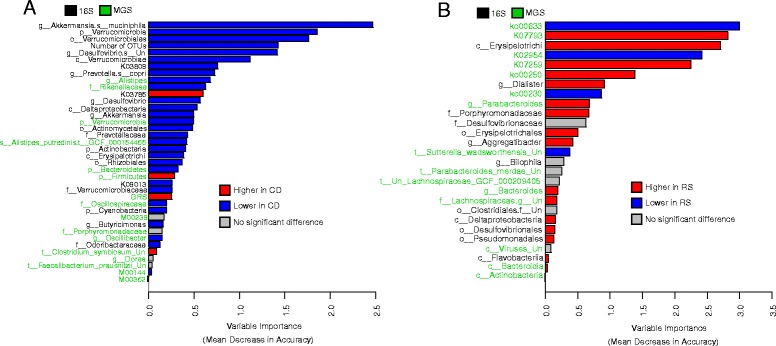
Variable importance of features in combined random forest models for **a** disease state classification and **b** treatment response classification. Red and blue are used to indicate which class has a higher mean standardized relative abundance. Features that did not significantly differ (*P* ≥ 0.05) between classes based on a two-tailed Mann-Whitney-Wilcoxon test are indicated in gray. Features in black and green font indicate 16S rRNA gene and shotgun metagenomics sequencing origins, respectively. “Un” stands for “Unclassified” when used in taxa names

Although comparing RF model accuracies allows individual datasets to be evaluated, it does not allow the relative importance of features across datasets to be evaluated. To this end, we next compared the relative importance of the overall top features by running RF models using the top three features from the significant datasets for both CD state (Fig. 3a) and treatment response (Fig. 3b). The combined model for disease state classification performed with high accuracy (accuracy = 78.9%, *P* < 0.001), but notably, this was lower than the 16S genera alone. In contrast, the combined model for treatment response classification performed better than the independent datasets (accuracy = 94.4%, *P* < 0.001). As expected, many of these features in both models are highly correlated (Additional file 1: Figure S8 and S9); nonetheless, this approach yielded several useful results. Firstly, *Akkermansia muciniphila* was ranked as the most important feature for classifying disease state, followed by Verrucomicrobia and Verrucomicrobiales, which represent the phylum and order of *A. muciniphila* respectively. Number of OTUs was ranked fourth among these features, whereas GRS and other MGS-derived features were ranked lower. Notably, 29/37 (78%) of the microbial features in this model were at lower relative abundances in CD patients compared to controls. The top three features for classifying treatment response in the combined model were ko00633, the nitrotoluene degradation pathway, K07793, the putative tricarboxylic transport membrane protein, and Erysipelotrichi (the class containing the family Erysipelotrichaceae). Unlike for the combined disease model, MGS-derived functions were among the most highly ranked features (all six MGS functions are within the first eight top features).

### Validating the best 16S disease feature rankings in an independent cohort

We validated the rankings of a subset of the 16S features (excluding the unclassified species in *Desulfovibrio*) used in the combined model for disease state by training a new model based on these features on the RISK cohort [8], a large previously published dataset, that consisted of 16S data for 731 biopsy samples (444 CD and 287 CN) after processing. The goal of this analysis was to determine if the top features for classifying disease state would have similar relative importance ranks across both cohorts. Only 16S sequencing of biopsy samples is available in this dataset and so we excluded GRS and the MGS features from this analysis. The new RF model based on this subset of features and trained on the RISK dataset was highly significant although less accurate than what we observed in our data (accuracy = 73.2%, *P* < 0.001). However, the relative ranking of these features was substantially different within the BISCUIT and RISK cohorts (Fig. 3a and Additional file 1: Figure S10). The top features in the RISK model were the class Erysipelotrichi, the phylum Actinobacteria, and the KO K09013. In addition, 8/21 16S features were not statistically different between CD and control patients (M-W-W test *P* ≥ 0.05). In particular, both *Desulfovibrio* and *Akkermansia* did not significantly differ between CD and control patients within the RISK cohort.

## Discussion

In this study, we have classified treatment-naïve pediatric CD patients by both their disease state and treatment response with high accuracies with many different microbial datasets. Since these microbial profiles were taken from intestinal biopsy samples, the main challenge of this study was to identify true microbial markers above the background of human DNA in the MGS data. Although we could identify microbial markers by generating much higher sequencing depth than is usual, the interpretation of analyses of this data come with the caveat that important rare taxa may have been below the detection threshold. For instance, although the RF models based on the MGS datasets were less accurate classifiers of disease state, this likely was impacted by the fact that the most informative genera in the 16S data were undetected in many MGS samples. This observation suggests that the discrepancy between the 16S and MGS taxonomic classification accuracies could be partially due to a relatively greater taxonomic depth of 16S sequencing, currently cost-prohibitive for MGS of biopsy samples, which enabled rarer taxa to be identified.

Since the 16S data does not face these challenges, interpreting the analyses based on these datasets is more straightforward. Indeed, many of the top features in the significant 16S datasets used to classify disease state (see Additional file 4) have previously been associated with IBD. For instance, sulfur-reducing species within the *Desulfovibrio* genus have previously been positively linked to another form of IBD—ulcerative colitis [48], and Mottawea et al. recently showed the importance of hydrogen sulfide producers in colonic CD [49]. However, we found *Desulfovibrio* to be negatively associated with CD in our data, which could highlight a difference in microbiota between these two forms of IBD or merely reflect the different sampling strategies (stool, biopsy and MLI) between IBD studies to date. We also found *Akkermansia muciniphila* to have lower relative abundance in CD patients’ biopsies, which has been previously observed [50]. The top 16S-inferred KOs are also related to functions previously associated with CD symptoms. The lower proportion of K09013 (Fe-S cluster assembly ATP-binding protein) in CD patients is interesting to find since intestinal inflammation in general has been associated with the breakdown of Fe-S clusters [51]. Similarly, both K03809 (tryptophan repressor binding protein) and K03785 (3-dehydroquinate dehydratase I), which is involved in tryptophan and other amino acid biosynthesis, in CD patients could be interesting markers since lower serum tryptophan levels has previously been associated with CD [52, 53]. However, in this analysis, these markers were both at higher levels in the unexpected direction (K03809 was lower in CD and K03785 was higher in CD).

The top MGS-identified features for classifying disease state also include several previously identified markers. The genus *Alistipes* is a known producer of short-chain fatty acids (SCFAs) [54]. This genus was at lower relative abundance in CD patients, which could be related to lower levels of certain SCFAs that have long been a hallmark of IBD [55–57]. In addition, although several key taxa identified by 16S sequencing appeared to be below the detection threshold in the MGS samples, both *Alistipes* and *Oscillibacter*, which has previously been negatively associated with CD [58], were not identified in the 16S data. The absence of these informative taxa is likely related to how certain lineages cannot be identified with high-resolution based on 16S sequences. This difference highlights a trade-off in the MGS taxonomic results: improved taxonomic resolution at the cost of lower sensitivity, which has been discussed elsewhere [59]. The identification of the MGS-identified KEGG module M00144, which is involved in ATP synthesis, as being informative for classifying disease state is also interesting since IBD patients are known to have lower levels of intestinal ATP [60].

Similar to the RF models for disease state, many of the top features for classifying treatment response agreed with previous studies (see Additional file 5). For instance, the top 16S genus, *Dialister*, was at higher abundance in RS patients, which is consistent with previous work [61]. Similarly, the bacterial family Erysipelotrichaceae has been linked to human health in several ways [62]. Although this taxon was not ranked highly, it is the only family within the top 16S-identified order, Erysipelotrichales, to pass pre-processing cut-offs. This order is found at higher relative abundance in RS patients. Erysipelotrichaceae are particularly of interest since they have been shown to decrease in abundance in CD patients given EEN therapy [63] and species within this family are positively linked to inflammation [64].

Several of the top MGS-identified KEGG functions also consistent with past work. The pathway ko00633, nitrotoluene degradation, has previously been identified as the most distinguishing pathway between EEN-treated CD patients and healthy controls [65]. Similarly, microbial glutathione and purine biosynthesis have previously been positively and negatively associated with Crohn’s disease respectively [57]. In our dataset, the pathway ko00250, glutamate and other amino acid metabolism, was found at higher relative abundance in RS patients whereas ko00230, purine metabolism, was found at lower relative abundance in RS patients. In addition, both the genera *Parabacteroides* and *Bacteroides* have previously been found at higher abundance in CD patients at the time of surgical resection who remain in remission [66], which is the same direction we find here. Our previous work in this cohort determined that *Sutterella wadsworthensis* is unlikely to be involved in IBD pathogenesis [67]. However, since this species was one of the best predictive features for treatment response and was found at lower abundance in RS patients, it may still be clinically relevant. Although these results indicate that future CD treatments could be informed by the presence of these and other microbial markers, further work will be required to disentangle which markers are predictive of response to specific treatments.

The findings of *Akkermansia muciniphila* and the order and phylum (Verrucomicrobiales and Verrucomicrobia, respectively) that contain this species as the top three features for classifying disease state, highlights the importance of this taxon in our dataset. High levels of *A. muciniphila* in donor’s stool has recently been found to be a strong predictor of remission in ulcerative colitis patients undergoing fecal microbiota transplantation treatment [68]. This finding taken together with our and others’ observation of lower *A. muciniphila* abundance in CD patients suggests that this species is a useful biomarker for gut health. Similarly, the relative importance of alpha-diversity compared to genetic risk was also shown in this combined model. This finding illustrates the importance of microbial features in CD development, as compared with the weak contribution of genetic markers for CD development and the influence of the inherited variants on microbiome composition [69]. The top MGS-identified features largely performed worse than the 16S-identified functions in the combined RF model for classifying disease. One interesting exception is the genus *Alistipes* (and its corresponding family Rikenellaceae).

In the combined RF model for treatment response, it is notable that MGS-identified functions were the most informative features. This observation could indicate that major metabolic shifts in the microbiome could be more informative for predicting treatment response than the presence of particular taxa, which is consistent with past results indicating that functions shift more consistently than taxa in CD patients [57]. Interestingly, functions were only found to be more informative for classifying patients by treatment response, and not by disease state. However, it is possible that with higher sequencing depth, MGS-identified features may have been more informative. Note that patients’ GRS were not significantly different between RS and NR samples, which is consistent with a recent study indicating that the genetic contributions to CD susceptibility are largely independent from the genetic contributions to CD prognosis [70].

Ideally the combined RF model trained on the top features from our cohort would also have been tested on the validation cohort. However, due to technical differences across the studies, such as different sampling protocols and different 16S variable regions sequenced, the same model cannot be implemented for both datasets. In addition, variation in pathophysiology due to geography as well as differential microbial profiles due to different distributions of patient age and sex across the cohorts could also result in differences in predictive markers across the two cohorts. This issue highlights that additional work in this area is needed to facilitate the comparison of microbiome datasets from different studies. Nonetheless, the independent validation cohort enabled the ranking of features within the combined model for disease state to be evaluated. The ranking of these features did differ in this cohort although the number of OTUs, Verrucomicrobiales, and Verrucomicrobia remain within the top six features (Additional file 1: Figure S10). However, the genera *Desulfovibrio* and *Akkermansia* were not significantly different between CD and control patients within the RISK samples, which highlights the issue of comparing predictive features across different cohorts. Unfortunately, we were unable to validate the ranking of the top features for classifying treatment response on an independent dataset since there is no paired 16S and MGS dataset with adequate sample size available to our knowledge.

## Conclusions

Here, we have integrated human genetic data with 16S and MGS intestinal biopsy data to classify CD patients by disease state and treatment response for the first time. We found genera identified from 16S data to be the best classifiers of each outcome. One possible explanation for why 16S data was found to have higher performance than the MGS data could be that it enables much higher read depth for taxonomic assignment. This increased depth allows rare taxa to be identified, which was the case for the top 16S-identified genera. The biological importance of rare taxa in CD pathogenesis warrants further consideration, and indeed, rarity may prove an important bias in culture-based studies of the IBD microbiome. Although we found alpha-diversity to be a clear marker for disease state, GRS was relatively less informative. This result is perhaps not surprising since microbial shifts are likely causally related to disease onset, although the direction is unclear. In contrast, GRS has been developed as a metric for assessing disease risk at any point in a patient’s life, including well before onset, but has not been of great influence in predicting onset or treatment stratification [1]. The multi-genomics machine learning approach presented in this study could be extended in the future to other diseases and to other data types such as transcriptomics and metabolomics to better understand the relative importance of each of these features. These models will provide new insights into the multifactorial nature of CD and help highlight cohort-specific as well as fundamental contributors to disease pathophysiology and may result in novel signatures to predict and guide personalized treatments.

## Additional files

Additional file 1: Random forest model summaries. (PDF 2413 kb)

Additional file 2: Sample sequencing coverage and metadata (XLSX 49 kb)

Additional file 3: Each dataset’s classification accuracy (XLSX 65 kb)

Additional file 4: Ranking of variable importance in significant models to identify important features. (XLSX 226 kb)

Additional file 5: The most informative features in each significant dataset (XLSX 151 kb)
