# Supplementary material for: Multi-omics differentially classify disease state and treatment outcome in pediatric Crohn’s disease
Source: Microbiome. 2018 Jan 15;6:13. doi: 10.1186/s40168-018-0398-3 (PMC5769311; doi:10.1186/s40168-018-0398-3)
Supplement: Supplementary file 1 — Random forest model summaries. (PDF 2413 kb) [file 40168_2018_398_MOESM1_ESM.pdf]

Supplementary Figures and Tables

- **Supplementary Figure 1**: Stacked bar-chart showing the relative abundance of microbial classes across the metagenomic sequencing data.
- **Supplementary Figure 2**: Stacked bar-chart showing the relative abundance of bacterial classes across the 16S rRNA gene sequencing data.
- **Supplementary Figure 3**: Barplots comparing random forest model accuracies based on non-rarified centered log-ratio transformed and rarified 16S rRNA gene taxa abundances.
- **Supplementary Figure 4**: Boxplots of genetic risk scores and the number of observed OTUs between Crohn's disease and control patients.
- **Supplementary Figure 5**: Distribution of variable importance for 16S rRNA gene-identified genera disease classification.
- **Supplementary Figure 6**: Boxplots of the relative abundance of the top 16S rRNA gene sequencing-identified genera for classifying disease state compared between sequencing technologies.
- **Supplementary Figure 7**: Boxplots of genetic risk score and alpha-diversity between responders and non-responders to treatment.
- **Supplementary Figure 8**: Pairwise Spearman correlation coefficients between features in the combined disease random forest.
- **Supplementary Figure 9**: Pairwise Spearman correlation coefficients between features in the combined treatment response random forest.
- **Supplementary Figure 10**: Features ranked by their relative importance for classifying disease state in the RISK validation cohort.
- **Supplementary Table 1**: Table of demographic and phenotypic characteristics of paediatric patients.
- **Supplementary Table 2**: Table of phenotypic characteristics and treatments of paediatric patients.

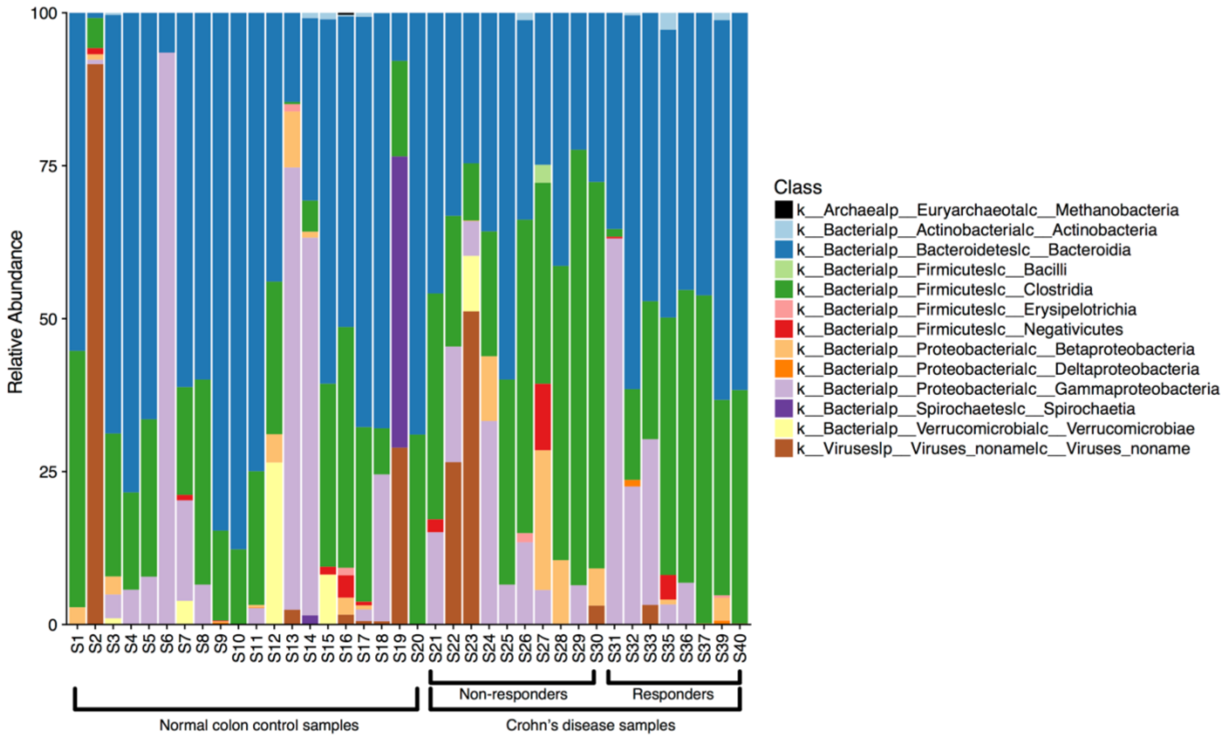

Supplementary Figure 1: Stacked bar-chart showing percentages of classes across metagenomic samples. Note the presence of archaea and viruses, which are absent in the 16S data (Supplementary Figure 2). Also, note the high prevalence of viral DNA in several samples. The metadata groupings of these samples are indicated at the bottom.

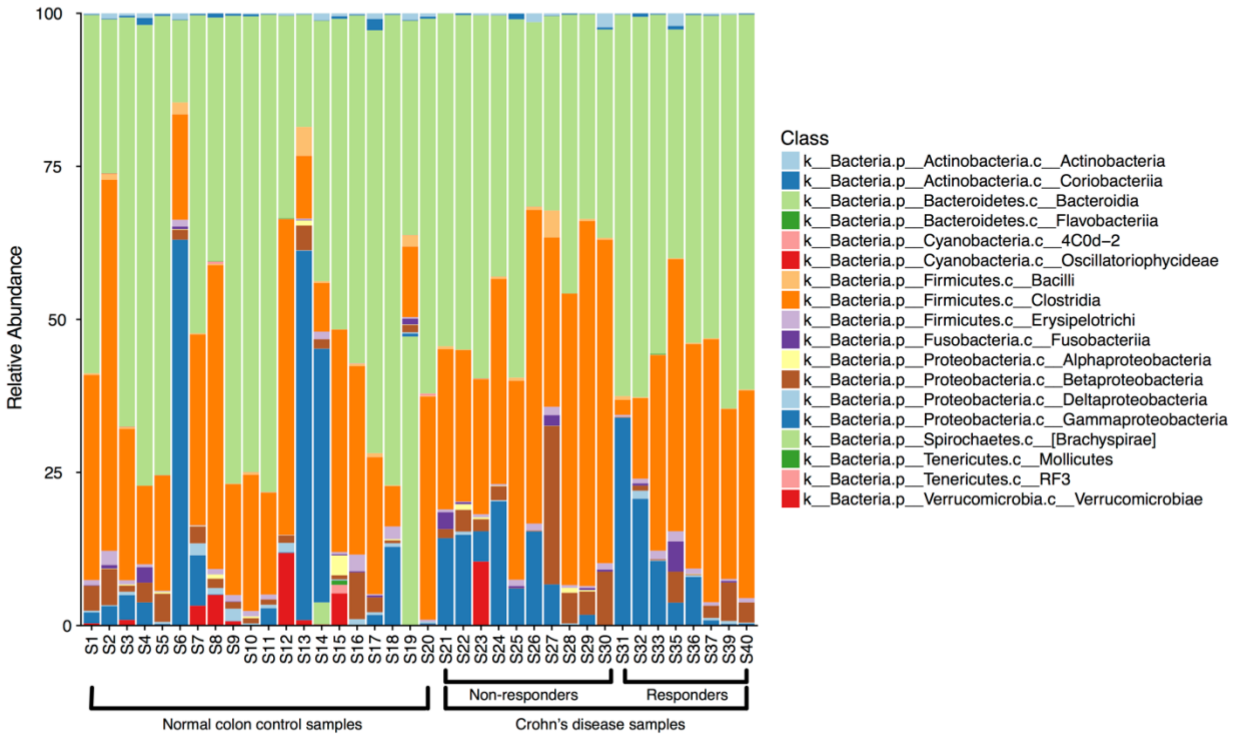

Supplementary Figure 2: Stacked bar-chart showing percentages of classes across 16S rRNA gene sequencing samples. Colours were chosen to help with discerning different taxa; however, several taxa have the same the colour so the taxa ordering should be considered when interpreting this figure. The metadata groupings of these samples are indicated at the bottom.

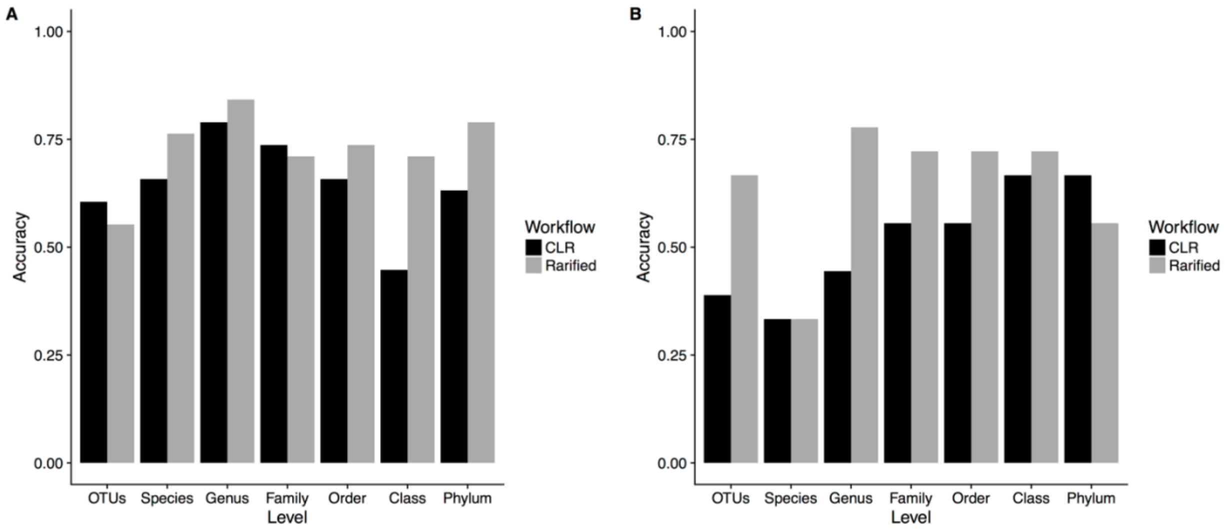

Supplementary Figure 3: Comparison of taxonomic dataset accuracies either transformed by centered log-ratio or rarified. The accuracies of random forest models trained on taxonomic datasets for each taxonomic level to classify patients by (A) disease and (B) treatment response are shown.

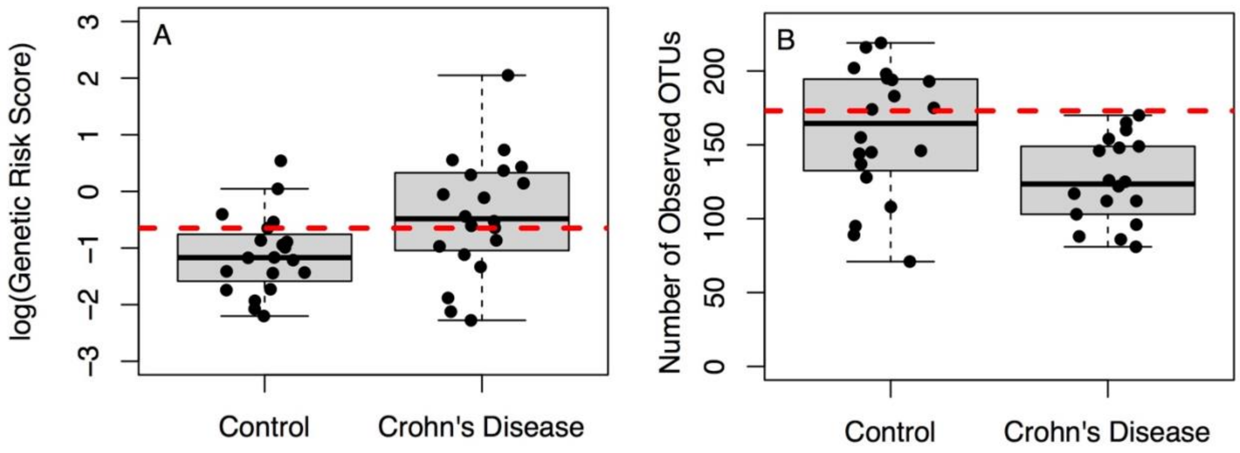

Supplementary Figure 4: Boxplots of (A) genetic risk scores on a natural log scale and (B) the number of observed OTUs, which is a measure of alpha-diversity. Samples are shown as black points. Red dotted lines correspond to the best cut-offs to distinguish the classes. There are 20 control and 18 Crohn's disease samples shown in each panel. The Mann-Whitney-Wilcoxon test were used to compare these groups since it is a non-parametric test whose main assumption is only that the data-points be independently distributed.

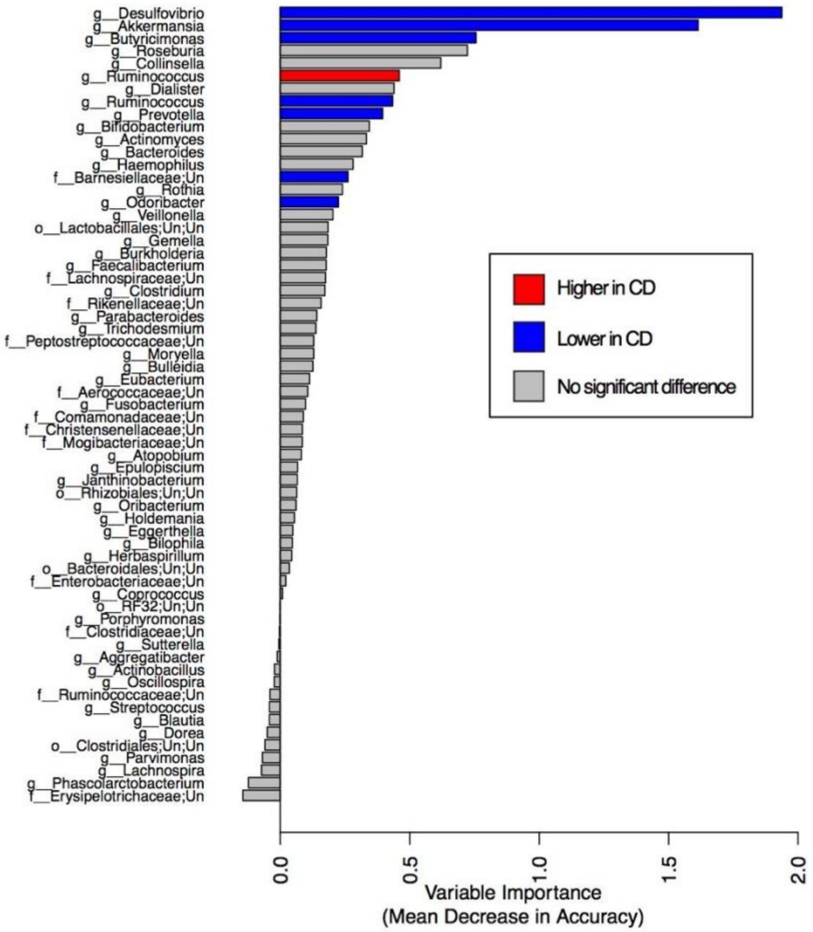

Supplementary Figure 5: Genera identified through 16S rRNA gene sequencing ranked by their importance for classifying disease state. Features that significantly differed (raw P < 0.05) between Crohn's disease (CD) and healthy colon control patients based on a two-tailed Mann-Whitney-Wilcoxon are indicated in red (if more abundant in CD patients) or blue (if lower in CD patients). Features that did not differ between the two classes are shown in grey.

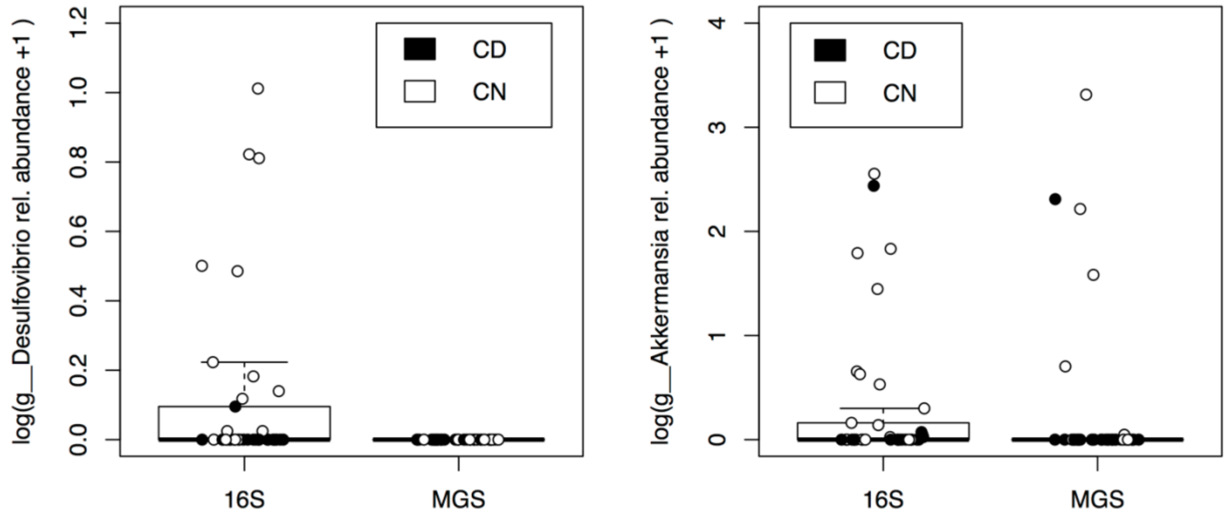

Supplementary Figure 6: Boxplots of the natural log relative abundance of the genera (A) *Desulfovibrio* and (B) *Akkermansia* for both sequencing technologies. These two genera had the highest variable importance in the most accurate disease classification random forest based on 16S rRNA gene sequencing data. Crohn's disease (CD) patients are indicated by black points and healthy colon controls (CN) are indicated as white points. A pseudocount of 1 was added to each sample's relative abundance since the log of 0 cannot be taken. Note that *Desulfovibrio* was absent in all metagenomic samples.

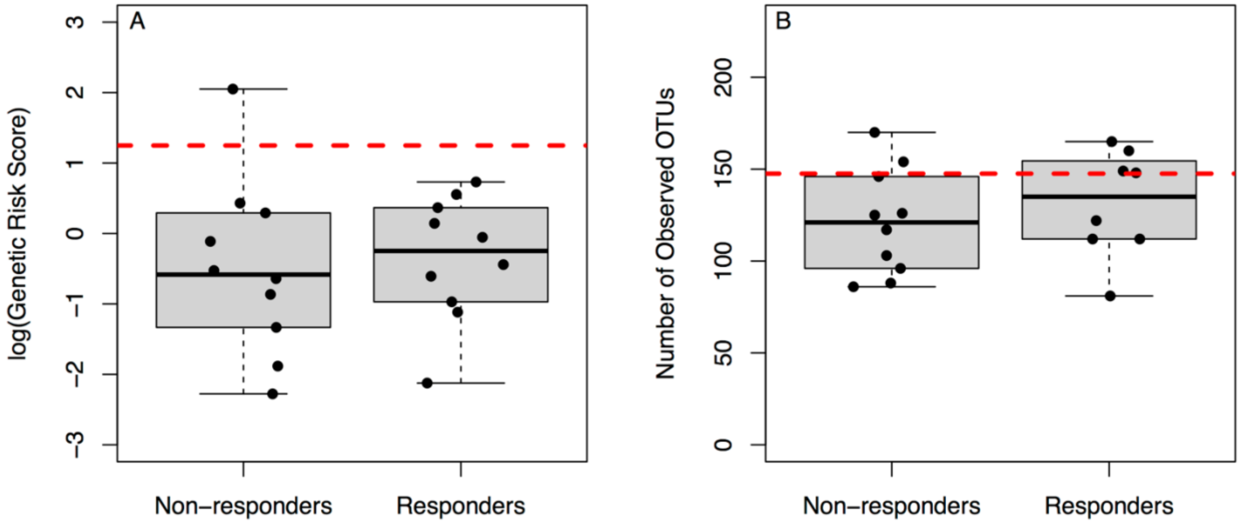

Supplementary Figure 7: Boxplots of (A) natural log genetic risk scores (GRS) and (B) number of observed OTUs (# OTUs) based on Crohn's disease patients' response to treatment. Both metrics did not significantly differ between non-responders and responders based on one-tailed Mann-Whitney-Wilcoxon tests (GRS: W=42, P=0.736; # OTUs: W=47, P=0.282). One-tailed tests were conducted based on the hypothesis that responders would have lower GRS and increased # OTUs.

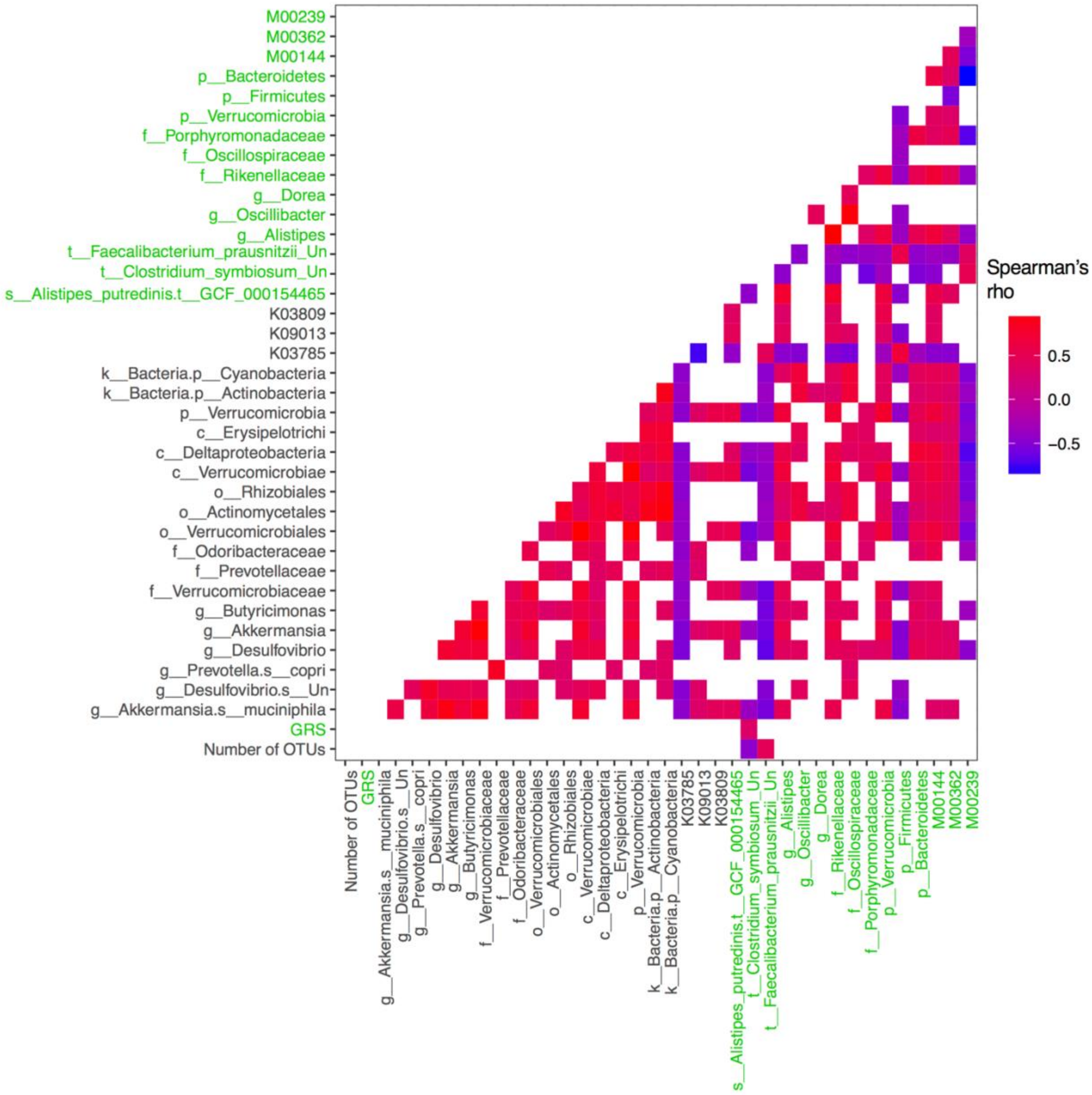

Supplementary Figure 8: Heatmap of Spearman correlation coefficients for features in the combined disease random forest model that were significantly correlated (P < 0.05). Metagenomics-identified feature names are coloured green. Only the bottom triangle is shown for simplicity. OTU: Operational Taxonomic Unit, GRS: Genetic Risk Score, Un: Unclassified.

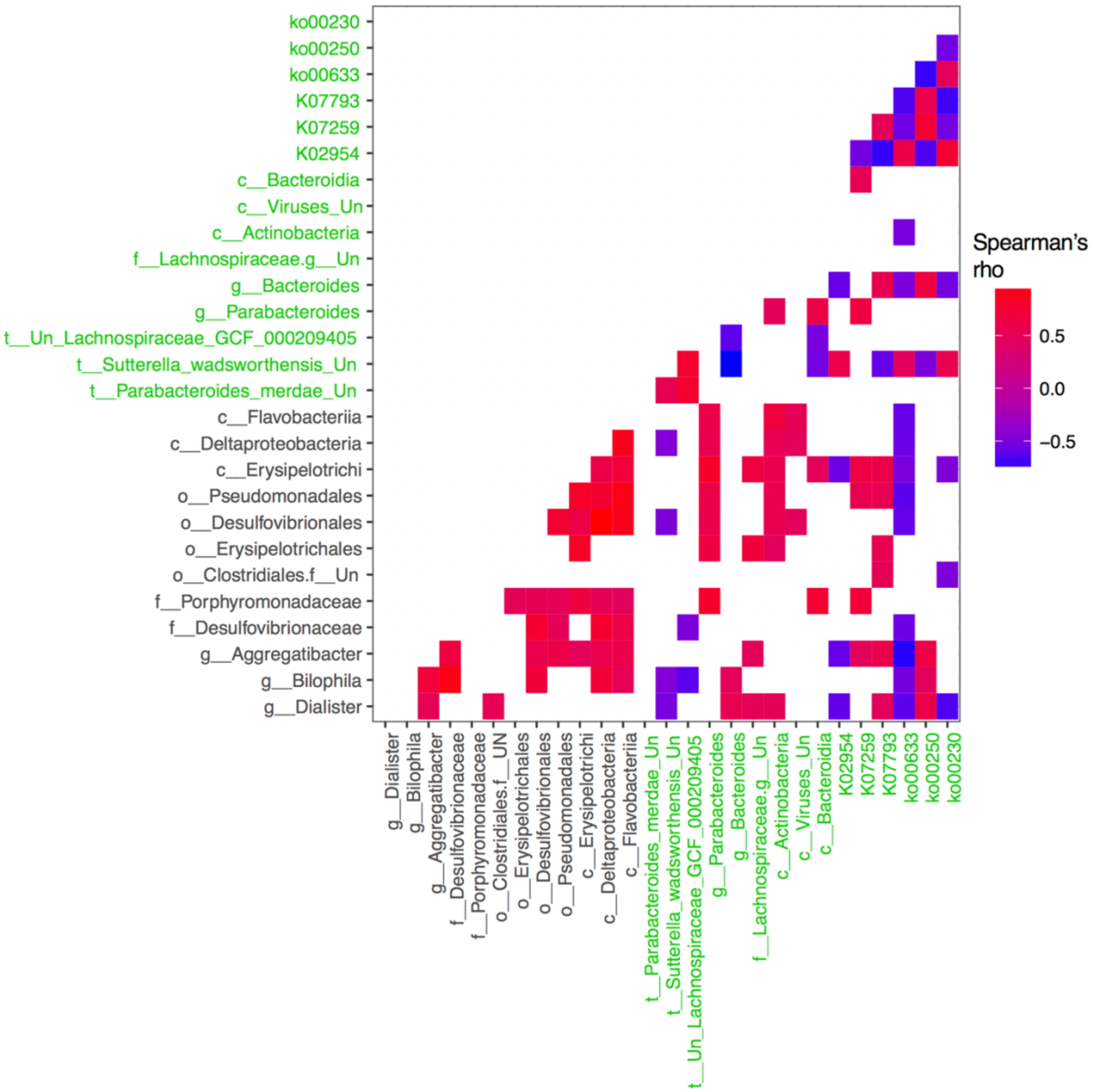

Supplementary Figure 9: Heatmap of Spearman correlation coefficients for features in the combined treatment response random forest model that were significantly correlated (raw P < 0.05). Metagenomics-identified feature names are coloured green. Only the bottom triangle is shown for simplicity. “Un” stands for “Unclassified”.

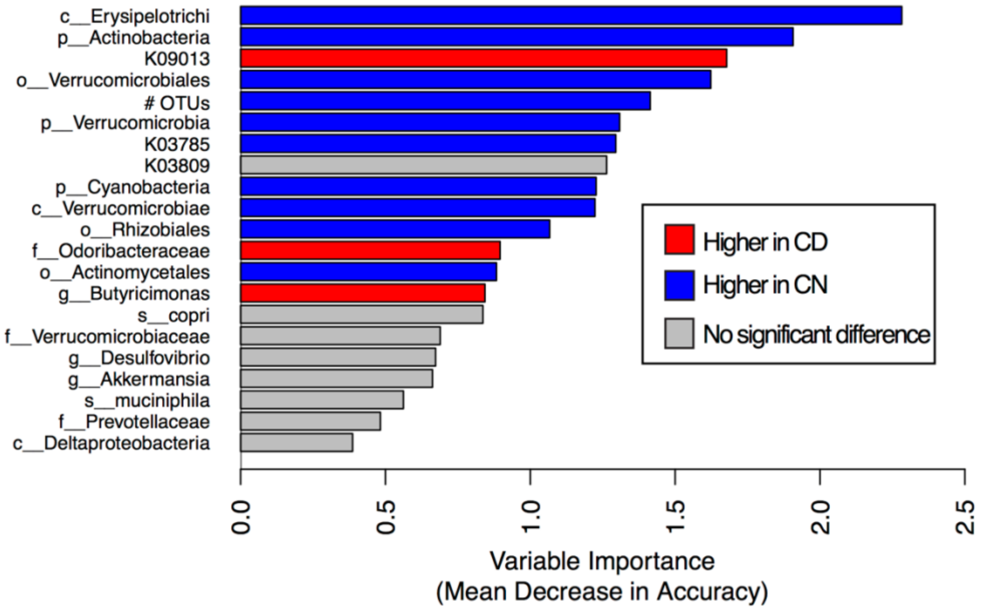

Supplementary Figure 10: The top features from the disease state combined random forest model ranked by their variable importance in a new model trained on the RISK validation data. Note that the 16S-identified unclassified species in *Desulfovibrio* was excluded since it was not present in the RISK data. All MGS features (including the genetic risk scores) were excluded from this analysis since MGS biopsy data was not available for this cohort. Features are coloured by whether they are significantly more abundant (raw $P < 0.05$) in Crohn's disease (CD; red) or normal colon control (CN; blue) samples, or not significantly different based on a two-tailed Mann-Whitney-Wilcoxon test.

ID	Sex	Age	BiopsySite	IBDDiagnosis
S1	Male	14.4	Rectum	Normal colon control
S2	Female	12.0	4 Sigmoid, 2 Caecum	Normal colon control
S3	Male	11.9	Sigmoid	Normal colon control
S4	Male	15.3	Rectum	Normal colon control
S5	Female	13.1	Caecum	Normal colon control
S6	Male	15.0	4 Sigmoid, 2 Caecum	Normal colon control
S7	Male	14.0	Sigmoid	Normal colon control
S8	Female	8.6	Sigmoid	Normal colon control
S9	Male	13.7	Rectum	Normal colon control
S10	Male	14.8	Sigmoid	Normal colon control
S11	Male	15.3	Sigmoid	Normal colon control
S12	Female	8.6	Sigmoid	Normal colon control
S13	Male	14.4	Sigmoid	Normal colon control
S14	Male	11.7	Rectum	Normal colon control
S15	Male	15.4	Sigmoid	Normal colon control
S16	Male	7.6	Rectum	Normal colon control
S17	Male	11.5	Sigmoid	Normal colon control
S18	Female	14.2	4 Sigmoid, 2 Caecum	Normal colon control
S19	Male	10.7	5 Sigmoid, 2 Caecum	Normal colon control
S20	Male	13.4	Sigmoid	Normal colon control
S21	Male	12.9	Sigmoid	Crohn's disease
S22	Female	8.0	Sigmoid	Crohn's disease
S23	Male	10.8	Sigmoid	Crohn's disease
S24	Male	16.3	Sigmoid	Crohn's disease
S25	Female	10.2	Rectum	Crohn's disease
S26	Male	14.5	Sigmoid	Crohn's disease
S27	Male	11.8	Sigmoid	Crohn's disease
S28	Female	11.9	Descending	Crohn's disease
S29	Male	15.2	Sigmoid	Crohn's disease
S30	Male	12.2	Sigmoid	Crohn's disease
S31	Male	14.8	Sigmoid	Crohn's disease
S32	Female	12.5	Descending	Crohn's disease
S33	Male	14.2	Rectum	Crohn's disease
S34	Male	15.0	Caecum	Crohn's disease
S35	Male	7.6	Descending	Crohn's disease
S36	Female	11.4	Sigmoid	Crohn's disease
S37	Male	14.2	Sigmoid	Crohn's disease
S38	Male	14.1	Caecum	Crohn's disease
S39	Male	11.2	Rectum	Crohn's disease
S40	Male	15.5	Rectum	Crohn's disease

Supplementary Table 1: Demographic and phenotypic characteristics of children with Crohn’s disease (highlighted in light blue) and normal colon controls from the BISCUIT study.

ID	Granulo-matous	Paris at Diagnosis	Primary Induction	Secondary within 150 days	Time from Diagnosis	Maximum Maintenance Agent(s)
S21	Yes	L3, B1	EEN	Steroid	133	Thiopurine
S22	Yes	L3, B1	EEN	Steroid	77	Thiopurine
S23	Yes	L3+L4a/b, B1p, OFG	EEN	Steroid	18	Methotrexate
S24	Yes	L3+L4a, B1	EEN	Steroid	18	Thiopurine
S25	Yes	L2, B1	Mesalazine	Steroid	109	Thiopurine
S26	No	L2, B1	Mesalazine	EEN	63	Thiopurine
S27	Yes	L2, B1	EEN	Steroid	29	Thiopurine
S28	Yes	L3+L4a, B1	EEN	Steroid	13	Methotrexate
S29	Yes	L2, B1	EEN	Prednisolone	62	Thiopurine
S30	Yes	L2+L4a, B1	Mesalazine	EEN	106	Mesalazine only
S31	Yes	L3+L4a/b, B1	Steroid			Thiopurine
S32	No	L3+L4a, B2	EEN & Steroid			Thiopurine
S33	Yes	L3+L4a, B1, OFG	Steroid			Thiopurine
S34	Yes	L3, B2	EEN & Steroid			Thiopurine + Biological
S35	Yes	L3+L4a, B1	Mesalazine			Thiopurine
S36	Yes	L3, B1	EEN			Thiopurine
S37	Yes	L3+L4a, B1	EEN			Thiopurine
S38	Yes	L3, B1	EEN			Methotrexate
S39	Yes	L3, B2	EEN			Thiopurine
S40	Yes	L3+L4a, B1	EEN & Steroid			Missing data

Supplementary Table 2: Phenotypic characteristics and treatments of children with Crohn's disease from the BISCUIT study. Non-responders to treatment are the red rows while responders to treatment are the white rows. The EEN treatment was Modulen.
